# First typology of cacao (*Theobroma cacao* L.) systems in Colombian Amazonia, based on tree species richness, canopy structure and light availability

**DOI:** 10.1371/journal.pone.0191003

**Published:** 2018-02-05

**Authors:** Juan Carlos Suárez Salazar, Marie Ange Ngo Bieng, Luz Marina Melgarejo, Julio A. Di Rienzo, Fernando Casanoves

**Affiliations:** 1 Universidad de la Amazonia, Facultad de Ingeniería, Campus Porvenir Calle 17 Diagonal 17 con Carrera 3F - Barrio Porvenir, Florencia, Caquetá, Colombia; 2 Universidad Nacional de Colombia, Facultad de ciencias, Departamento de Biología, Laboratorio de Fisiología y Bioquímica vegetal, Bogotá, Colombia; 3 CIRAD, UR Forêts et Sociétés, CIRAD Campus International de Baillarguet, Montpellier Cedex 5 France; 4 CATIE: Centro Agronómico Tropical de Investigación y Enseñanza, CATIE, Turrialba, Cartago, Costa Rica; 5 Facultad de Ciencias Agropecuarias, Universidad Nacional de Córdoba, Av. Valparaiso s/n, Ciudad Universitaria, Córdoba, Argentina; Wageningen University, NETHERLANDS

## Abstract

**Aim and background:**

We present a typology of cacao agroforest systems in Colombian Amazonia. These systems had yet to be described in the literature, especially their potential in terms of biodiversity conservation. The systems studied are located in a post-conflict area, and a deforestation front in Colombian Amazonia. Cacao cropping systems are of key importance in Colombia: cacao plays a prime role in post conflict resolution, as cacao is a legal crop to replace illegal crops; cacao agroforests are expected to be a sustainable practice, promoting forest-friendly land use.

**Material and methods:**

We worked in 50 x 2000 m^2^ agroforest plots, in Colombian Amazonia. A cluster analysis was used to build a typology based on 28 variables characterised in each plot, and related to diversity, composition, spatial structure and light availability for the cacao trees. We included variables related to light availability to evaluate the amount of transmitted radiation to the cacao trees in each type, and its suitability for cacao ecophysiological development.

**Main results:**

We identified 4 types of cacao agroforests based on differences concerning tree species diversity and the impact of canopy spatial structure on light availability for the cacao trees in the understorey. We found 127 tree species in the dataset, with some exclusive species in each type. We also found that 3 out of the 4 types identified displayed an erosion of tree species diversity. This reduction in shade tree species may have been linked to the desire to reduce shade, but we also found that all the types described were compatible with good ecophysiological development of the cacao trees.

**Main conclusions and prospects:**

Cacao agroforest systems may actually be achieving biodiversity conservation goals in Colombian Amazonia. One challenging prospect will be to monitor and encourage the conservation of tree species diversity in cacao agroforest systems during the development of these cropping systems, as a form of forest-friendly management enhancing sustainable peace building in Colombia.

## Introduction

Cacao is a commercial crop of great importance worldwide, with the production of 4,251 thousand tons of beans in 2016, on 9.9 million hectares and affecting the livelihoods of 40–50 million people [1]. Seventy percent of cacao crops are cultivated in agroforest systems, associated with trees (shades trees) and/or with annual and perennial crops [2].

In Latin America, 1.5 million hectares of cacao are cultivated, of which 70% are cultivated under shade trees in agroforest systems [2]. Cacao crops are native to Latin America, but the main production zone is now Africa: the annual production in Latin America in 2016 was 777 thousand tons of beans (18.3% of world cacao production), well below cacao production in Africa (with 3,074 thousand tons of beans [1]). In Latin America, the main producers are Ecuador and Brazil (261 and 230 thousand tons of beans respectively [1]). In the other countries of Latin America, cacao production was significantly reduced with the arrival of Monilia disease or Frosty Pod Rot [3].

Specifically in Colombia, in 2016, cacao cropping systems were an economic activity occupying 173,016 ha, with the production of 56,785 tons of beans. Now, cacao is considered as the “cultivo para la paz”[4], i.e. a crop to replace illegal cropping systems. For this reason, the department of Caquetá in Colombian Amazonia increased its area from 555 ha in 2007 to 1,350 ha in 2016 [5].

In addition, in the post-conflict context, cacao crops will have greater importance for Colombian Amazonia and for the country as a whole in the future, and especially for the economic development and well-being of the farmers located in the former postconflict zones [4]. Cacao agroforests are also expected to be a sustainable practice, promoting forest-friendly land use in the region of the Colombian Amazonian forest systems [6]. The Colombian forest currently provides a habitat for 10% of world diversity [7]. This biodiversity may be endangered in a post-conflict context (cacao agricultural intensification, increase in timber extraction) [6]. Cacao agroforestry may be a way to incorporate conservation and agronomic production within the challenge of safeguarding Colombian forest diversity.

However, until now, the potential of Colombian cacao agroforest systems in terms of biodiversity conservation had yet to be described in the literature. It therefore appeared important to procure some reliable knowledge of these cacao cropping systems.

In this context, the purpose of our work was to describe the different cacao cropping systems developed in the region of Caquetá in Colombian Amazonia, through a typology of cacao agroforests. The first step towards better management of such agroforest stands is to describe them precisely, and especially to take into account their variability by defining different types of stands. This step corresponds to a classic typological approach, which may better encompass the variability and the functioning of complex systems [8]. This first stage of description enables an assessment of the potential of such systems, and also provides an opportunity for sustainable management policies, with the long-term objectives of (i) optimizing the productive potential of cacao agroforest systems in Colombia and more generally in Latin America, and (ii) integrating biodiversity conservation in these systems as a great opportunity for biodiversity-friendly development.

Many typologies of agroforestry systems have been developed in the literature. Six general types of stand structure are known: full sun cacao (monospecific plantation), cacao under specialized shade, cacao under diversified shade, cacao under productive shade, cacao under rustic shade and a sequential agroforest arrangement [9–11]. These types of agroforest structure have been reported in Central America [12], South America [13] Africa [14,15] and Asia [16].

The amount of light transmitted to cacao trees is an important criterion differentiating the agroforest types [17,18], with the full sun system receiving the greatest amount. The amount of light transmitted to cacao trees directly affects cacao tree growth and yield, given that the optimum radiation transmitted for good ecophysiological development of cacao trees is 400 μmol m^-1^ s^-2^ [19]. This amount depends on the interception of solar radiation by canopy shade trees, and therefore on the density and the spatial structure of shade trees. Management of the shade provided by trees, in relation to the radiation transmitted to the cacao understorey, is a critical variable in cacao production. Shade also affects the temperature, humidity and vapour pressure deficit, among other parameters related to the ecophysiological performance and production of cacao [20,21]. Thus, quantifying the amount of light for the crop in the understorey of agroforest systems is a prerequisite for understanding the impact of shade on the productivity of such systems [22,23]. It appears interesting to integrate variables related to the amount of light transmitted in typologies of agroforest stands.

In this study, in addition to variables related to biodiversity, we built a typology of cacao agroforest systems taking into account, (i) variables related to the spatial organisation of associated shade trees, i.e. computing the Clark Evans index in each plot; (ii) and variables related to light availability in each plot; these variables were computed from field measurements, and others were simulated using two models: Shademotion and SExI-FS. The typology was based on 50 agroforest plots set up in cacao plantations in Colombian Amazonia. In each plot, we measured variables related to tree species composition, stand vertical structure, but also variables related to the spatial organisation of individuals and simulated variables related to light availability and distribution using two models. We then used a principal components analysis and cluster analysis to build a typology of the study plots, using a matrix composed of the different measured and simulated variables. Lastly, we discuss the findings in relation to tree species richness (as a proxy for conservation) and light availability for cacao trees (as a proxy for potential production capacity) in the different types of our typology.

## Materials and methods

### Study area and plot selection

The study was set up in the Bajo Caguán zone of the department of Caquetá-Colombia, in Colombian Amazonia (0°31'06.5"N 74°23'05.0"W), where the climate is warm-humid, characteristic of the ecosystem of tropical humid forests, with an annual average rainfall of 3,800 mm, 1,700 hours of sunshine per year, an average temperature of 25.5°C and an air relative humidity of 84%. To our knowledge, the soil type, which is known to be diverse in the Amazon region, has not been specifically characterised in that very isolated post-conflict region. The mean altitude is 280 m.a.s.l. We investigated cacao agroforest structures in plantations along the river Caguán, among the stands of Santafé del Caguán, Camelias, Remolinos del Caguán and Guamo, specifically in the following villages: El Guamo, Sabaleta, Caño Santo Domingo, Caño Negro, Cuba, Remolinos, Las Claras, Palmichales, Cristales. The geographical coordinates of the different plots are provided in the supplementary information. This zone is considered as a deforestation front in Colombian Amazonia. Indeed, these agroforest plantations were set up in former tropical primary forest areas. This zone is also a prioritary zone for biodiversity conservation, as it is very close to the national park of Chiribiquete, in the heart of Amazonia.

We selected fifty cacao agroforest plantations, belonging to producers of the “Asociación de productores de cacao de Remolinos del Caguán y Suncillas CHOCAGUAN”, which is the only cacao producer organisation in this zone. The 50 cacao agroforest plantations were selected randomly from the diversity of plantations existing within the framework of that association. In each plantation, we set up a 2,000 m^2^ plot (100 m x 20 m) in the centre, which we verified to be representative of the stand characteristics of the plantation as a whole (cacao density, density of associated trees). The plantations were part of farms with a mean area of 110 ha, where the cacao agroforest area was about 4 ha. The plots displayed contrasting management characteristics (species composition of the associated shade trees or plants, shade management, plantations of different ages).

### Variables studied for the typology

#### Composition and tree species richness

We characterised the plant diversity associated with cacao trees in the studied cacao agroforest plots, in terms of associated trees, palms and Musaceae. We took differently into account (i) trees and (ii) palms and Musaceae, in order to account for the different levels of shade intensity that they provided to the cacao trees in the understorey [24]. In comparison to palms and Musaceae, trees are generally the tallest in these agroforests, forming the top of the canopy and providing the highest level of shade, a shade that is more homogeneous and less close to the cacao trees. Palms and Musaceae belong to the intermediate vegetation layers, closer to the cacao trees. Trees are therefore expected to provide shade that is less localized, not as close and less deep than the shade of palms and Musaceae. Moreover, in terms of management, Palms and Musaceae provide a more temporal shade as they are more frequently used (hence cut) than trees by the farmers.

Specifically for trees, we characterized the species richness of the associated trees in each plot. We determined the species of each tree in each plot and the abundance of each tree species in each plot. For the associated palms and Musaceae, we only noted their density, in each plot.

#### Vertical and horizontal structure

Vertical distribution was ascertained in each plot by separating associated trees into 3 height layers: high (25–35 m), intermediate (9–24 m) and low (1–8 m). We took into account trees identified taxonomically and with a DBH (Diameter at Breast Height) greater than or equal to 10 cm. The DBH was measured for each tree, and we also evaluated its basal area, and the shape and the area of its crown based on three diameters from its projection on the ground. A novel approach in this study was that we took into account the horizontal spatial structure of the trees, as a key element of the description of these agroforest systems [24]. In order to characterize the horizontal spatial organisation of individuals, we noted the (x, y) positions of each tree in each plot, using a tape measure. It should be pointed out that the measurements only took into account the associated trees. We only took into account the palms and Musaceae when characterising the area of the crown.

#### Transmitted radiation

In order to have an evaluation of the amount of light transmitted to the cacao trees in the understorey of the shade trees in each plot, we took hemispherical photographs (using a Nikon Coolpix 4500, with an FC-E8 hemispherical lens, Nikon Co. Japan). The photographs were taken at the four cardinal points of each tree that formed the shade canopy layer, considering the coordinates of the given tree in each plot. Each hemispherical photograph was analysed with Gap Light Analyzer software (GLA [25]). In addition, we measured the intensity of Photosynthetically Active Radiation (*PAR*, in μmol m^-2^ s^-1^) using an AcuPAR LP-80 sensor (Decagon Devices Inc., Pullman, WA, USA). For each tree, PAR was measured above and in the four cardinal points below each crown with 2 sensors.

### Data analysis

#### Species richness, vertical and horizontal spatial structure

We computed tree species richness (number of species in each plot), but we also computed tree species richness and the Shannon and Simpson diversity indices [26]. We also computed the Importance Value Index (IVI) [27] from the relative abundance RA (%), relative dominance RD (%) and relative frequency RF (%) of each tree species. For the species *i*, the *IVI*_*i*_ was computed following the formula:

$$IVI_{i}=RA_{i}+RD_{i}+RF_{i}$$

Where

$$RA_{i}=\frac{N_{i}}{N};\;RD_{i}=\frac{BA_{i}}{BA_{T}};\;RF_{i}=\frac{NP_{i}}{NP_{T}}$$

Where N_i_ is the number of individuals of species i, N the total number of individuals BA_i_ is the basal area of species i, BA_T_ is the basal area of all the species, NP_i_ is the number of plots where species i is present, NP_T_ the total number of plots.

In order to explain the physiognomic complexity of the agroforest structure, we computed the Physiognomic Predominance Index PPI [28], which depends on the relative abundance RA (%), relative dominance RD (%) and the relative cover RC (%) of each individual tree found in the plot.

For species i, the PPI_i_ was computed using the formula:

$$PPI_{i}=RA_{i}+RD_{i}+RC_{i}$$

Where

$$RC_{i}=\frac{CA_{i}}{CA_{T}}$$

and CA_i_ is the crown area cover of the individual of species i, and CA_T_ the crown area of all the individuals.

In order to characterize the horizontal spatial distribution of the associated trees, we used the nearest neighbour index *R*_*CE*_ [29]. This index determines the spatial dispersion of shade trees in the canopy. It describes the relationship between, (i) the average of the distances between each tree and its nearest neighbour in the study plot and, (ii) the same average of the distances on a null hypothesis of the random distribution of trees. The interpretation was: *R*_*CE*_ >1 is regularity in the spatial distribution of individuals; *R*_*CE*_ = 1 is random; RCE<1 is aggregation. This index is a measurement of the deviation from a null hypothesis of a random distribution of trees, and is a very useful tool for detecting structural changes at plot level.

#### Transmitted radiation

Firstly, based on the hemispherical photograph, the Leaf Area Index (LAI) was computed per plot. This was proportional to the relationship between the leaf area and the corresponding area of the plot.

Secondly, for each shade tree, the fraction of light intercepted was computed as the quotient between the intensities of PAR above and below the given tree crown; the fraction of light intercepted PAR per plot was obtained from the individual value of each tree. We therefore built a two-dimensional map of PAR distribution per plot. The coefficient of light extinction (Kn) per plot was calculated by:

$$K(x,y)=e^{\left(PARt(x,y)/PARi(x,y))/LAI(x,y\right)}$$

where (*x*,*y*) is the location of each point (tree) in the plot, PARt the PAR intensity under shade trees, *PARi* is the intensity of PAR above shade trees and LAI is the leaf area index [30].

Lastly, we used two models:

1. Shademotion 4.0 [31] to calculate the fraction of the average of shade hours (SH) and shade area (SA) in each agroforest plot, from variables such as the position of each tree in each plot (x,y), crown characteristics (type, width, height, opacity), trunk height. Shademotion is a software modelling the shade projected by shade trees on flat, horizontal or inclined land (http://shademotion.net/)).
2. Spatially individual-based Explicit Forest-Simulator SExI-FS [32] to calculate the degree of canopy openness in each agroforest plot, from variables such as the position of each tree in each plot (x,y), DBH, total height of the tree and crown characteristics (radius, depth and curve). SExI-FS is a simulator that focuses on tree-tree interactions in an agroforest system. (http://www.worldagroforestry.org/output/sexi-fs-spatially-explicit-individual-based-forest-simulator)).

### Typology of cacao plots

Our objective was to define types of agroforest systems, and our typology was based on a matrix of 28 variables characterising the following in each plot: diversity (4 variables), composition (6), vertical spatial structure (9), horizontal spatial structure (3), and transmitted radiation (4). We carried out a Principal Components Analysis (PCA) on the obtained matrix, from which the principal components were selected according to the criterion of the mean eigenvalue. A cluster analysis was carried out using the adopted components. The number of clusters was decided using the criterion of the gDGC test [33] with a significance level of 0.05. In order to identify which original variables better characterized the defined types found in the cluster analysis, we undertook univariate analyses of variance (using the LSD Fisher test (p <0.05)) for each of the 28 variables using the defined types as classification criteria. The analyses of principal components, clusters and variance were done using the InfoStat program [33]. The FactoMineR package [34] was used to obtain the graphs of the hierarchical clustering on a factorial plane made up of the first two principal components (hierarchical clustering on the factor map). The PCA graphs were produced using the Ade4 package [35] of R version 3.4.0 [36].

## Results

### The typology of cacao plots in Colombian Amazonia

**Fig 1:**
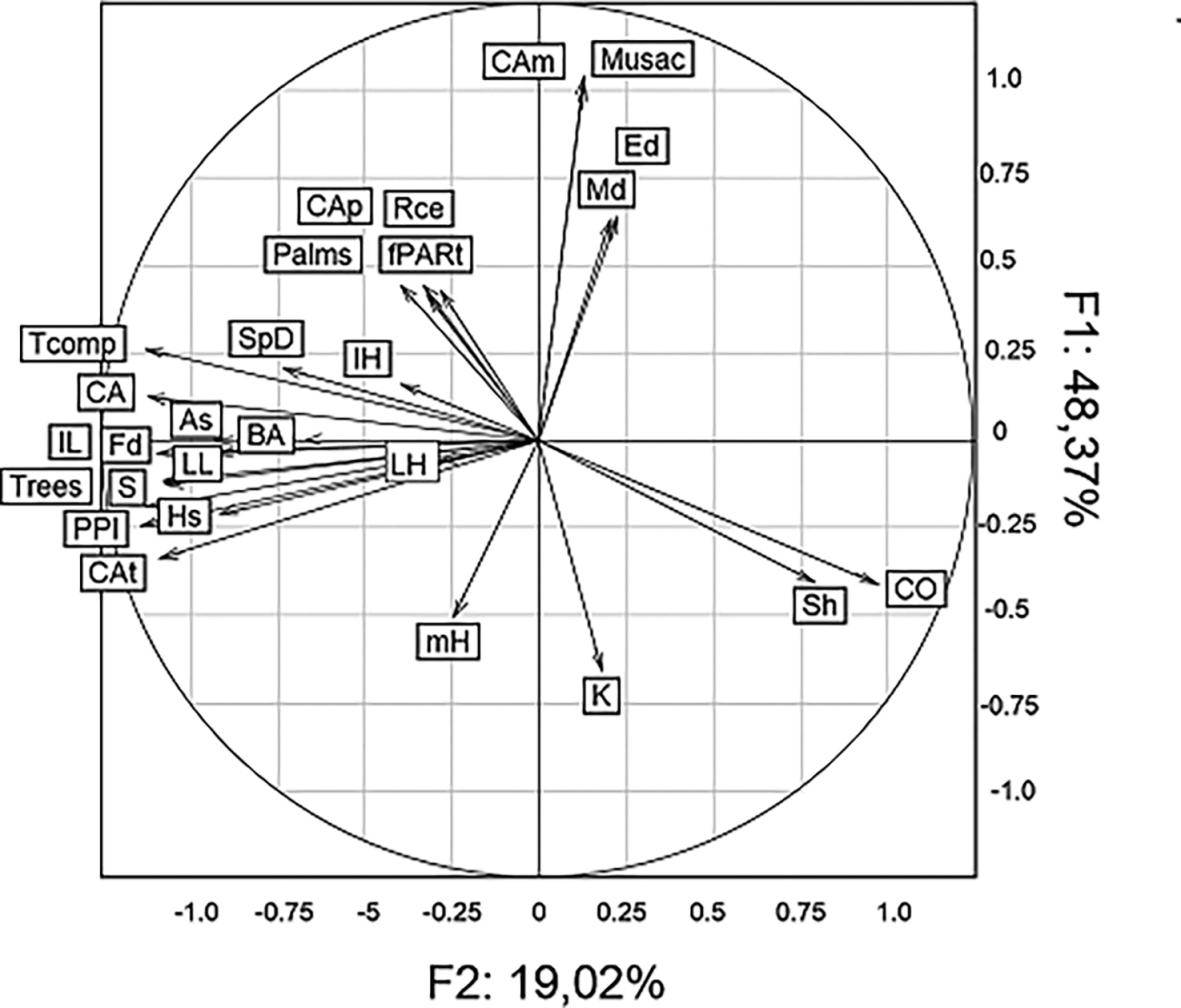
Representation of the PCA carried out on the matrix of 28 variables characterised in each plot: 4 variables for diversity, 6 for composition, 9 for vertical spatial structure, 3 for horizontal spatial structure, and 4 for transmitted radiation. The abbreviations for each variable are explained in Table 1.

We present here the types of agroforest systems found, based on a matrix of 28 variables characterised in each plot (see Table 1 for the different variables). From the PCA carried out on the matrix of the measured, computed and simulated variables, the first eight components were chosen with 85% of the original variability represented. The PCA analysis identified the most associated variables in each type (Fig 1): the first axis explained 48.37% of the variance and separated the “complex diversified multistrata” type from the three others, which were significantly less complex. This separation was attributed to its complexity in terms of tree species richness, composition and spatial organisation. The second axis (19.2%) separated the “high density of Musaceae” type from the other types, and this was due to its high Musaceae and palm composition.

**Table 1:** The 28 variables characterised in each parcel of the studied agroforest plots and their mean values in each type of the typology built. Values represent the mean ± standard deviation. P-value shows the differences between types of cacao agroforests in Colombian Amazonia.The four types are: complex diversified multistrata (CDM), low diversity with regular trees (LDR), low diversity with clustered trees (LDC), and high density of Musaceae (HDM). We have: 4 variables for diversity, 6 for composition, 9 for vertical spatial structure, 3 for horizontal spatial structure, and 4 for transmitted radiation. (LSD Fisher test significant at p <0.05).

Component	Variable	abbreviations	Units	CDM (13 plots)	LDR (10 Plots)	LDC (18 Plots)	HDM (9 Plots)	General	p-valor
Diversity	Species richness	S	Nb/plots	12.92±0.96a	5.86±0.89b	3.14±0.89b	3±1.49b	6.14±0.74	<0.0001
	Families	Fd	Nb/plots	9.83±0.61a	4.86±0.57b	2.86±0.57c	2.6±0.95c	4.98±0.51	<0.0001
	Shannon index	Sh		3.19±0.13b	3.85±0.12a	3.95±0.12a	3.04±0.21b	3.65±0.08	0.0011
	Simpson index (1-D)	SI		0.76±0.04a	0.76±0.04a	0.59±0.04b	0.6±0.07b	0.64±0.03	<0.0001
Number of individuals	Total	Tcomp	Nb/ha	302.5±17.68a	106.43±16.37c	54.29±16.37d	194±27.39b	141.43±16.77	<0.0001
	Trees	Trees	Nb/ha	278.33±16.39a	95±15.18b	47.86±15.18b	38±25.4b	114.49±15.8	<0.0001
	Palms	Palms	Nb/ha	24.17±8.49ab	4.29±7.86b	6.43±3.25b	54±13.15a	14.49±4.5	<0.0001
	Musaceae	Musac	Nb/ha		7.14±6.04b		102±10.11a	12.45±5.3	<0.0001
	Low layer	LL	Nb/ha	136,9±17,8a	47,84±7,42b	27,4±6,71c	32,5±13,14bc	62,73±8,71	<0.0001
	Intermediate layer	IL	Nb/ha	153,14±18,42a	48,31±10,76b	41,4±20,64b	37,5±21,42b	70,81±10,42	<0.0001
Structure									
Vertical	Basal area	BA	m^2^/ha	6,17±1,12a	3,74±0,82b	3,21±1,54bc	2,36±1,1c	3,94±0,68	0,045
	Height (mean)	mH	m	8.31±0.36a	6.8±0.36b	9.02±0.39a	6.73±0.6b	7.97±0.24	<0.0001
	Height low layer	LH	m	6.59±0.52a	6.8±0.36ab	5.63±0.49ab	4.53±0.81b	5.66±0.31	0,045
	Height intermediate layer	IH	m	9.94±0.73ab		10.57±0.68a	10.04±1.14ab	9.22±0.43	<0.0001
	Crown area (total)	CA	m^2^	626.60±38.06a	231.17±23.16c	274.17±38.35c	438.36±90.88b	348.82±31.01	<0.0001
	Trees	CAt	m^2^	577.89±48.21a	206.13±22.36b	246.18±37.97b	142.67±41.05b	291.56±29.96	<0.0001
	Palms	CAp	m^2^	48.71±17.29ab	9.83± 6.25b	27.99±15.93b	118.98±80.00a	34.88±10.77	<0.0001
	Musaceas	CAm	m^2^		15.21± 8.16b		176.71±49.10a	22.38± 9.11	<0.0001
	Physiognomic Predominance Index	PPI		12.53±0.79a	4.45±0.73b	4.62±0.73b	3.42±1.22b	6.12±0.65	<0.0001
Horizontal	Mean distance	Md	m	2.41±1.21b	3.3±1.12b	4.31±1.12b	8.73±1.87a	3.98±0.66	<0.0001
	Expected distance	Ed	m	2.35±1.06b	3.11±0.98b	3.4±0.98b	7.65±1.64a	3.5±0.58	0,0169
	Nearest neighbor index	R_CE_		1±0,03c	1,32±0,05a	0,9±0,03d	1,21±0,05b	1,12±0,04	<0.0001
Radiation	Transmitted radiation	fPARt	μmol m^-2^ s^-1^	680.4±60.1c	820.3±220.8c	1200.8±80.6b	1400.6±100.2a	1025.5±115.4	<0.0001
	Light extinction coefficient	Kn		4.41±0.64b	5.88±0.59b	2.89±0.59a	2.58±0.99a	4.36±0.39	<0.0001
	Shade area	SA	m^2^ ha^-1^	4836.67±69.72a	3833.43±161.16b	3627.65±274.95b	2799.20±61.13ab	3776.06±146.58	<0.0001
	Shade hours	SH	hr	325.57±24.55a	166.2±12.63b	167.9±22.73b	109.62±38.03b	204.27±15.48	0.0006
	Canopy openess	CO	%	31.42±1.49b	58.64±3.72a	67.93±5.26a	78.45±6.54b	59.11±6.68	<0.0001

**Fig 2:**
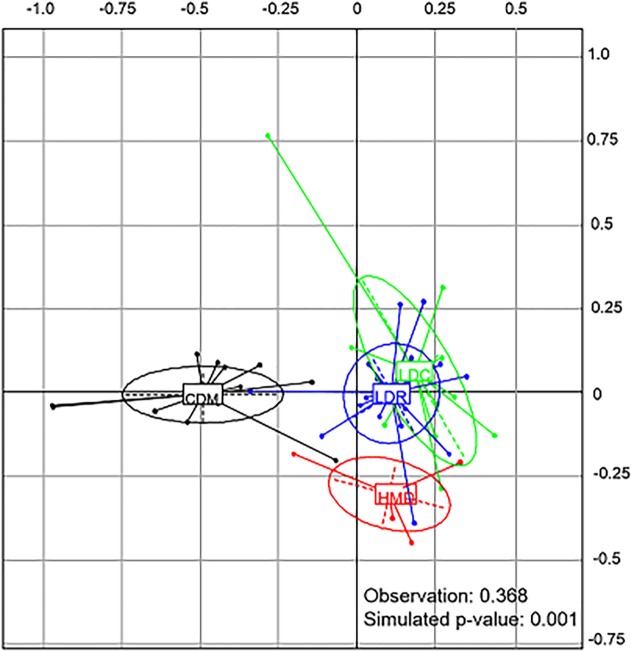
Representation of the hierarchical clustering on the factor map made on the selected principal components obtained from our matrix of 28 variables. The variables are for diversity (4 variables), composition (6), vertical spatial structure (9), horizontal spatial structure (3), and transmitted radiation (4). The cluster analysis defined 4 types: complex diversified multistrata (CDM), low diversity with regular trees (LDR), low diversity with clustered trees (LDC), and high density of Musaceae (HDM).

**Fig 3:**
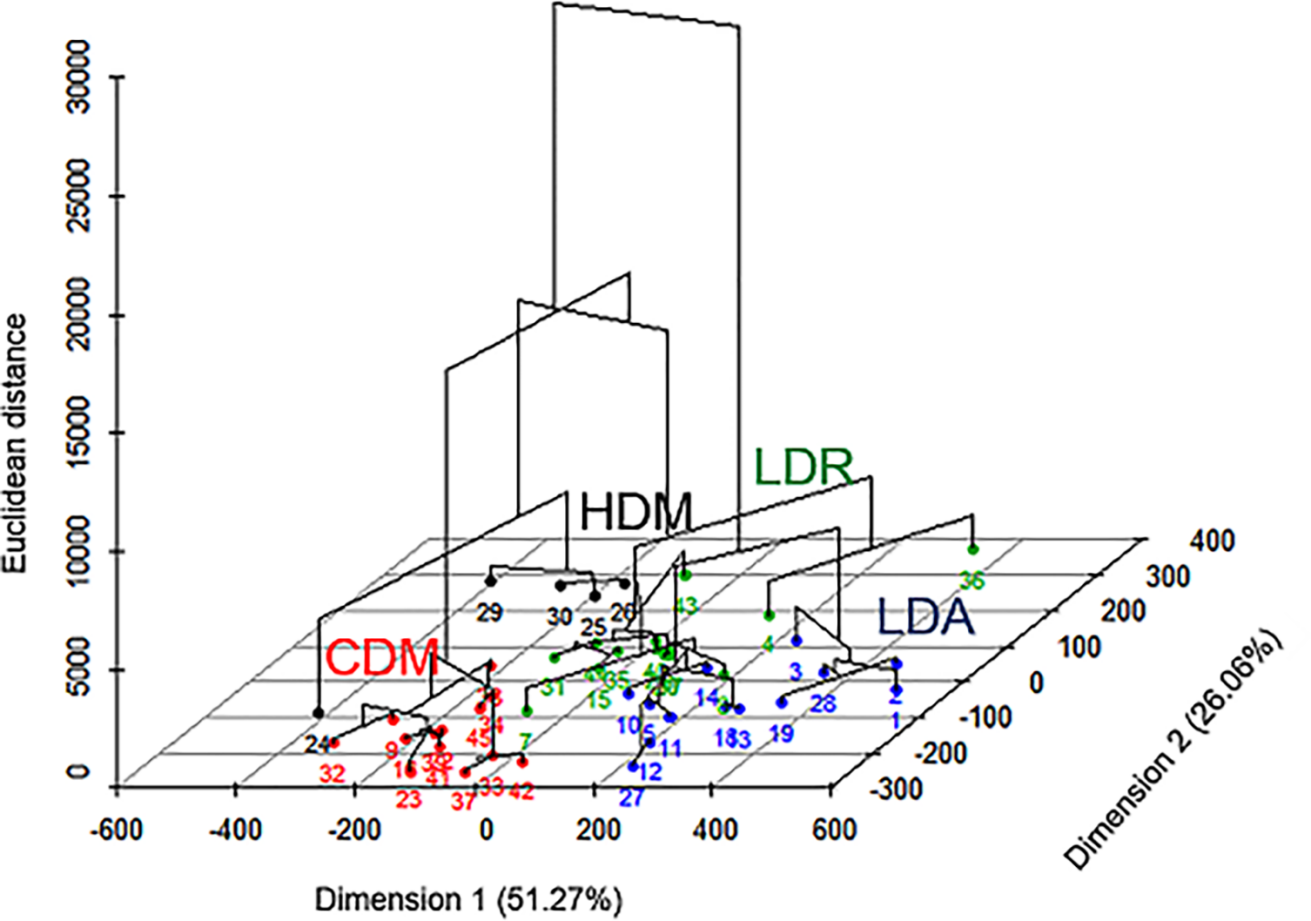
Representation of the types of our typology in a Cartesian plane made with the first two axes of the principal components analysis. The 4 types were: complex diversified multistrata (CDM), low diversity with regular trees (LDR), low diversity with clustered trees (LDC), and high density of Musaceae (HDM).

We found four types of agroforest plots (Fig 2): complex diversified multistrata (CMD), low diversity with regular trees (LDR), low diversity with clustered trees (LDC), and high density of Musaceae (HDM). Types LDR and LDC, both with a low diversity of tree species, were different because of the spatial organisation of the associated trees. Type HDM, also with a low diversity of tree species, was different because of the high density of Musaceae in the corresponding plots. The Monte Carlo test with permutations of the coordinates of each point (plot) indicated that the separation between the types was highly significant (P < 0.001) and explained 36.8% of the variance between the types (Fig 3).

#### Complex diversified multistrata (CDM), 13 plots

This type characterised cacao agroforest plots with the highest diversity compared with the other types of the typology: an average of 12.92±0.96 species in the 13 plots (see all the information related to the number of families and diversity indices in Table 1). In the 13 plots, there was an average of 302.5±17.68 individuals per hectare, of which 91.4% were trees and the rest palms (Table 1). In this type, there were no Musaceae. The composition of individuals in relation to the strata was characterised as: low, 136.9±17.8 individuals per hectare and intermediate, 153.14 individuals per hectare; these numbers of individuals per stratum were higher compared to the other types (Table 1). The basal area (6.17±1.12 m^2^ ha^-1^) was twice as large in comparison to the other types, with large trees (height and crown), reflected in a higher PPI (12.53). At horizontal level, the trees were close to each other, with an average distance of 2.41±1.21 m between trees and displayed a random spatial organisation (R_CE_ = 1). Concerning light availability for the cacao trees in the understorey, the average transmitted light fraction, fPARt, in the 13 plots was 680.4±60.1 μmol m^-2^ s^-1^, the average coefficient of light extinction, Kn, was 4.41±0.64, the average annual number of shade hours in this type was 325.57±24.55, and the degree of canopy openness was 31.42±1.49%.

#### Low diversity with regular trees (LDR), 10 plots

The diversity of species in this type was less than in the previous type: an average of 5.86 species in the 10 plots. In the 10 plots, there was an average of 106.43 individuals per hectare, including a low density of Musaceae (7.14 individuals per hectare). The composition of individuals in relation to the strata was characterised by: 47.84 individuals per hectare in the low stratum and 48.31 individuals in the intermediate stratum (Table 1). The basal area was also lower than in the previous type, 3.74±0.82 m^2^ ha^-1^ with a PPI value of 4.45±0.73. At horizontal level, the distance between trees was not significantly different from that in the previous type, with an average distance of 3.3±1.12 m, but the spatial organisation of the trees was regular in this type (R_CE_>1) (Table 1). Concerning light availability for the cacao trees in the understorey, fPARt in the 10 plots was 820.3±220.8 μmol m^-2^ s^-1^, the average coefficient of light extinction, Kn, was 5.88±0.59, the average annual number of shade hours in this type was 166.2±12.63, and the degree of canopy openness was 58.64±3.72%.

#### Low diversity with clustered trees (LDC), 18 plots

The diversity of species in this type was low, with an average of 3.14±0.89 species in the 18 plots. However, the diversity of species was not significantly different from the number of species in the previous type (apart from the Shannon index). In the 18 plots, there was an average of 54.29±16.37 individuals per hectare, and there were no Musaceae. The composition of individuals in relation to the strata was characterised by: 27.4±6.7 individuals per hectare in the low stratum and 41.4±20.64 individuals in the intermediate stratum (Table 1). The basal area was lower than in the first type, and not significantly different from the second type, with a value of 3.21±1.54 m^2^ ha^-1^, and with a PPI value of 4.62±0.73. At horizontal level, the distance between trees was greater, but not significantly different from the previous two types, with an average distance of 4.31±1.12 m. The real difference from the previous type was the aggregated spatial distribution of trees, with R_CE_ <1 (Table 1). Concerning light availability for the cacao trees in the understorey, fPARt in the 18 plots was 1200.8±80.6 μmol m^-2^ s^-1^, the average coefficient of light extinction, Kn, was 2.89±0.59, the average annual number of shade hours in this type was 167.9±22.73, and the degree of canopy openness was 67.93±5.26%.

#### High density of Musaceae (HDM), 9 plots

The diversity of species in this type was the lowest of the four types, with an average of 3±1.49 species in the 9 plots. The main difference from the other types was the high density of Musaceae, at 102±10.11 individuals per hectare, but we also found trees, at 38±25.4 individuals per hectare and palms at 54±13.15 individuals per hectare. In the 9 plots, there was an average of 194±27.39 individuals per hectare. The composition of individuals in relation to the strata was characterised by: 32.5±13.14 individuals per hectare in the low stratum and 37.5±21.42 individuals per hectare in the intermediate stratum (Table 1). The basal area was the lowest of the typology, at 2.36±1.1 m^2^ ha^-1^, with a PPI value of 3.42±1.22. At horizontal level, the distance between trees was the highest in the typology, with an average distance of 8.73±1.87 m. (Table 1). The spatial distribution of trees was regular, R_CE_>1. Concerning light availability for the cacao trees in the understorey, fPARt in the 9 plots was 1400.6±100.2 μmol m^-2^ s^-1^, the average coefficient of light extinction, Kn, was 2.58±0.99, the average annual number of shade hours in this type was 109.62±38.03, and the degree of canopy openness was 78.45±6.54%.

### Trees species richness in the cacao agroforest systems of Colombian Amazonia

Trees species composition varied in the different types of cacao agroforest systems. For the whole dataset, we found 40 families, 112 genera and 127 tree species (only 4% of the tree species sampled were not identified and not taken into account in this work). In relation to the different types of our typology, we found 70 species in the CDM type, 35 in the LDC type, 15 in the LDR type and 7 in the HDM type. In our dataset, 57 tree species were exclusive to the CDM type, 25 to the LDC type, 8 to the LDR type and 2 to the HDM type (Table 2). Table 3 shows the IVI and the PPI for the different tree species associated with the cacao trees in the agroforests studied: 30 species displayed 204.5% and 229.5% for IVI and PPI, respectively; they were well represented in the agroforest systems studied. In the “other” category (Table 3), there were 109 other tree species that were less represented in the agroforest systems (they can be found in the supplementary data). Generally, the species frequently found independently of the type of agroforest system were *Inga minutula*, *Sapium marmieri*, *Spondias mombim* L, *Hieronyma alchorneoides*, *Psidium guajava* L and *Swietenia macrophylla*, which accounted for 109% and 132% of the total IVI and PPI, respectively. Concerning the types of the typology, *Sapium marmieri*, *Hieronyma alchorneoides* and *Inga minutula* had the highest IVI for CDM, LDC and LDR, respectively. For PPI, it was *Sapium marmieri*, *Hieronyma alchorneoides* and *Inga minutula* for CDM, LDC and LDR, respectively (Table 3). For the HDM type, the most frequent species were *Inga minutula*, *Spondias mombim* L, and *Acalypha cf*. *stachyura Pax*.

**Table 2:** List of tree species within the different shade layers in each type of the typology built. The types are: complex diversified multistrata (CDM), low diversity with regular trees (LDR), low diversity with clustered trees (LDC), and high density of Musaceae (HDM).

Types	Layer	More frequent tree species	Exclusive tree species	
CDM (n = 13)	Low	*Cecropia ficifolia Warb*. *ex Snethl*.	*Anacardium cf*. *Parvifolium*	*Picramnia sp*.
		*Cecropia sciadophylla Mart*.	*Andira cf*. *multistipula Ducke*	*Piper aduncum L*.
		*Citrus reticulata Blanco*	*Apeiba aspera Aubl*.	*Piptocoma discolor (Kunth) Pruski*
		*Cordia alliodora (Ruiz & Pav*.*) Oken*	*Bellucia pentamera Naudin*	*Platymiscium stipulare Benth*.
		*Fabaceae sp1*	*Calyptranthes cf*. *lanceolata O*. *Berg*	*Pouteria cf*. *caimito (Ruiz & pav*.*) Randlk*
		*Hieronyma alchorneoides Allemão*	*Clitoria cf*. *javitensis (Kunth) Benth*.	*Rinorea sp*. *1*
		*Inga minutula (Schery) T*.*S*. *Elias*	*Coccoloba sp*. *1*	*Rollinia edulis Triana & Planch*
		*Psidium guajava L*.	*Colubrina glandulosa Perkins*	*Sapium sp*. *1*
		*Sapium eglandulosum Ule*	*Diplotropis martiusii Benth*.	*Siparuna guianensis Aubl*.
		*Sapium marmieri Huber*	*Eschweilera coriacea (DC*.*) S*.*A*. *Mori*	*Sorocea cf*. *pubivena Hemsl*.
		*Spondias mombim L*.	*Gloeospermum longifolium Hekking*	*Tabebuia sp*. *1*
			*Grias cf*. *peruviana Miers*	*Talisia cf*. *hemidasya Radlk*.
			*Hamelia patens Jacq*.	*Tetrathylacium macrophyllum Poepp*.
			*Himatanthus tarapotensis (K*. *Schum*.	*Theobroma bicolor Bonpl*.
			*ex Markgr*.*) Plumel*	
			*Inga sp*. *1*	
			*Isertia hypoleuca Benth*.	
			*Miconia trinervia (Sw*.*) D*. *Don ex Loudon*	
			*Palicourea sp*.*1*	
	Intermediate	*Acalypha cf*. *stachyura Pax*	*Annona sp*. *1*	*Palicourea lasiantha K*. *Krause*
		*Cecropia ficifolia Warb*. *ex Snethl*.	*Brosimum cf*. *lactescens (S*. *Moore) C*.*C*. *Berg*	*Pentagonia macrophylla Benth*.
		*Cecropia sciadophylla Mart*.	*Brownea macrophylla horn*. *ex Mast*.	*pentagonia sp*. *1*
		*Ficus insipida Willd*.	*Casearia javitensis Kunth*	*Protium cf*. *glaberscens Swart*
		*Hieronyma alchorneoides Allemão*	*Chimarrhis glabriflora Ducke*	*Sapium glandulosum (L*.*) Morong*
		*Inga minutula (Schery) T*.*S*. *Elias*	*Coccoloba mollis Casar*.	*Siparuna decipiens (Tul*.*) A*. *DC*.
		*Pentagonia macrophylla Benth*.	*Conceveiba sp*. *1*	*Solanum altissimum Benítez*
		*Sapium eglandulosum Ule*	*Croton matourensis Aubl*.	*Spondias*
		*Sapium marmieri Huber*	*Jacaratia digitata (Poepp*. *& Endl*.*) Solms*	*Theobroma glaucum H*. *Karst*
		*Spondias mombim L*.	*Miconia cf*. *floribunda (Bonpl*.*) DC*.	*Virola duckei A*.*C*. *Sm*.
		*Swietenia macrophylla King*	*Moraceae sp2*	*Xylopia cf*. *amazonica R*.*E*. *Fr*.
LDC (n = 18)	Low	*Citrus limon (L*.*) Osbeck*	*Alchornea latifolia Sw*.	*Sapium peruvianum Steud*.
		*Hieronyma alchorneoides Allemão*	*Alibertia sp*. *1*	*Schefflera morototoni (Aubl*.*)*
		*Inga cayennensis Sagot ex Benth*.	*Andira cf*. *multistipula Ducke*	*Maguire*, *Steyerm*. *& Frodin*
		*Inga minutula (Schery) T*.*S*. *Elias*	*Asteraceae sp*.*1*	*Tetragastris panamensis (Engl*.*) Kuntze*
		*Spondias mombim L*.	*Billia rosea*	*Urera caracasana (Jacq*.*) Gaudich*. *ex Griseb*.
		*Swietenia macrophylla King*	*Citrus sinensis*	
			*Condaminea sp*. *1*	
			*Crescentia cujete L*.	
			*Morinda citrifolia L*.	
			*Prunus accumulans (Koehne) C*.*L*. *Li*	
			*& Aymard*	
			*Rollinia cf*. *cuspidata Mart*.	
	Intermediate	*Beilschmiedia sp*.*1*	*Alchornea glandulosa Poepp*.	*Parkia cf*. *velutina Benoist*
		*Guarea fissicalyx Harms*	*Allophylus floribundus (poepp) Randlk*	
		*Hieronyma alchorneoides Allemão*	*Chrysophyllum sp*. *1*	
		*Inga minutula (Schery) T*.*S*. *Elias*	*Citrus nobilis Lour*	
		*Psidium guajava L*.	*Fabaceae sp2*	
		*Sapium marmieri Huber*	*Ficus sp*. *1*	
		*Swietenia macrophylla King*	*Inga sp*. *3*	
LDR (n = 10)	Low	*Acalypha diversifolia Jacq*.	*Allophylus sp*. *1*	
		*Cecropia latiloba Miq*.	*Bauhinia tarapotensis Benth*.	
		*Inga minutula (Schery) T*.*S*. *Elias*	*Campsiandra cf*. *steyermarkiana Stergios*	
		*Spondias mombim L*.	*Dialium guianense (Aubl*.*) Sandwith*	
		*Swietenia macrophylla King*	*Inga sp*. *6*	
	Intermediate	*Inga minutula (Schery) T*.*S*. *Elias*	*Grias neuberthii J*.*F*. *Macbr*.	
		*Sapium marmieri Huber*	*Micropholis guyanensis (A*.*DC*.*) Pierre*	
			*Tabebuia rosea (Bertol*.*) A*. *DC*.	
HDM (n = 9)	Low	*Psidium guajava L*.		
		*Spondias mombim L*.		
		*Swietenia macrophylla King*		
	Intermediate	*Albizia sp*. *1*		
		*Asteraceae sp*.*1*		
		*Enterolobium schomburgkii (Benth*.*) Benth*.		
		*Inga minutula (Schery) T*.*S*. *Elias*		
		*Spondias mombim L*.	.	

**Table 3:** Importance value index (IVI) and physiognomic predominance index PPI for the tree species in the study plot, and within the different types of the typology built. The 4 types are: complex diversified multistrata (CDM), low diversity with regular trees (LDR), low diversity with clustered trees (LDC), and high density of Musaceae (HDM).

	General		IVI				PPI			
Species	IVI	PPI	CDM	LDR	LDC	HDM	CDM	LDR	LDC	HDM
*Inga minutula (Schery) T*.*S*. *Elias*	33.63	51.54	21.62	25.23	63.6	152.91	21.81	20.88	65.67	114.94
*Sapium marmieri Huber*	19.51	19.8	27.2	16.29	20.56	0	18.01	7.88	11.81	0
*Spondias mombim L*.	18.73	19.92	19.75	17.05	20.37	62.51	15.7	10.73	10.85	35.56
*Hieronyma alchorneoides Allemão*	16.24	18.21	16.3	32.23	0	0	12.44	29.88	0	0
*Psidium guajava L*.	11.37	10.81	6.76	16.22	0	0	3.62	15.72	0	0
*Swietenia macrophylla King*	9.58	11.78	3.99	18.31	17.78	0	3.9	23.79	12.6	0
*Ficus insipida Willd*.	7.92	6.97	13.31	4.04	4.94	0	7.73	2.15	2.45	0
*Miconia cf*. *floribunda (Bonpl*.*) DC*.	6.21	8.94	1.31	0	0	0	0.82	0	0	0
*Acalypha cf*. *stachyura Pax*	5.81	5.7	3.44	6	8.84	34.59	2.8	2.71	4.57	16.63
*Cecropia ficifolia Warb*. *ex Snethl*.	5.42	5.22	11.15	0	0	0	8.32	0	0	0
*Cecropia sciadophylla Mart*.	5.29	6.45	10.48	0	0	0	11.92	0	0	0
*Ficus sp*. *1*	4.88	5.23	0	16.65	0	0	0	4.27	0	0
*Guatteria coeloneura Diels*	4.65	7.07	1.52	0	0	0	0.98	0	0	0
*Sapium eglandulosum Ule*	4.48	5.41	7.69	0	0	0	8.72	0	0	0
*Croton matourensis Aubl*.	4.41	5.03	10.87	0	0	0	3.65	0	0	0
*Cordia alliodora (Ruiz & Pav*.*) Oken*	4.28	4.37	1.88	2.08	0	0	1.59	1.3	0	0
*Cassia sp*. *1*	4.14	4.83	1.58	0	23.48	0	1.02	0	11.43	0
*Guarea fissicalyx Harms*	4.06	3.91	1.16	12.32	0	0	0.81	7.44	0	0
*Ceiba cf*. *samauma (Mart*.*) K*. *Schum*.	3.75	2.22	2.64	4.38	0	0	1.14	2.18	0	0
*Leonia crassa L*.*B*. *Sm*. *& A Fernandez*	3.41	2.83	1.72	9.21	0	0	0.8	3.63	0	0
*Vitex orinocensis Kunth*	3.05	2.69	2.13	2.14	6.89	0	1.38	1.48	4.63	0
*Beilschmiedia sp*.*1*	3.02	3.37	2.75	6.19	0	0	1.76	5.42	0	0
*Citrus limon (L*.*) Osbeck*	2.93	1.8	1.09	6.24	0	0	0.51	3.23	0	0
*Acalypha diversifolia Jacq*.	2.85	2.19	2.66	2.02	9.32	0	1.78	1.19	8.28	0
*Heliocarpus americanus L*.	2.81	2.57	2.37	6.2	0	0	1.41	3.2	0	0
*Jacaranda copaia (Bertol*.*) A*. *DC*	2.79	3.03	0	3.76	13.09	0	0	3.42	5.51	0
*Cecropia latiloba Miq*.	2.58	1.63	1.29	4.37	7.12	0	0.67	2.1	5.18	0
*Trema micrantha (L*.*) Blume*	2.36	2.24	3.56	2	0	0	3.74	1.12	0	0
Other	200.18	225.76	180.21	212.94	195.99	250.01	137.03	153.72	142.97	167.13

### Light availability in the different types

**Fig 4:**
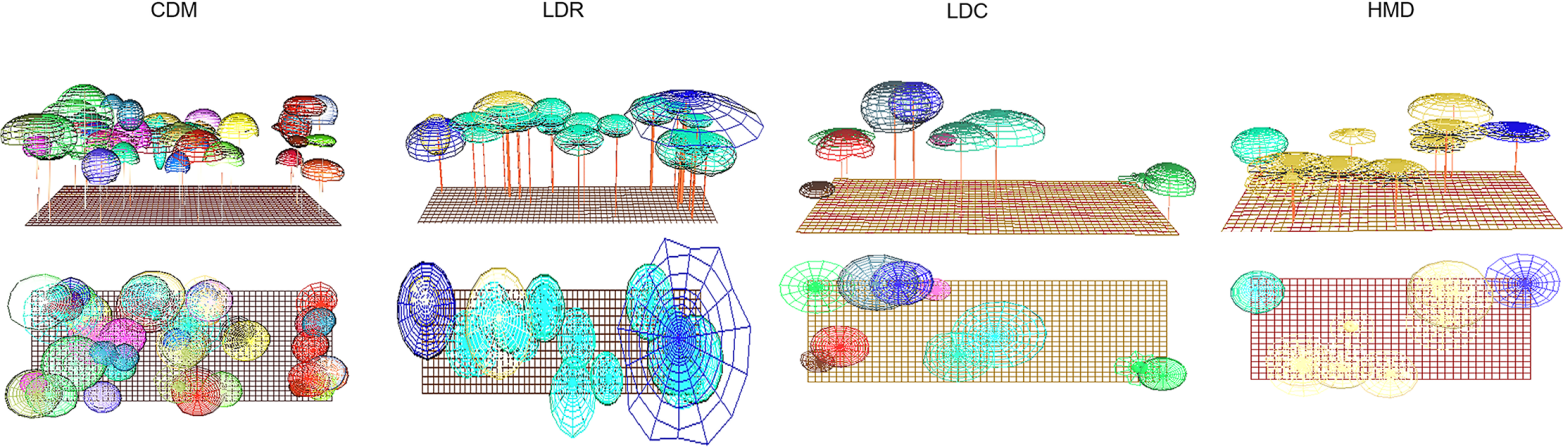
Simulated representation of the structure (horizontal and vertical) of a given plot illustrative of each type. The simulations were made with SExI-FS software. The four types were: complex diversified multistrata (CDM), low diversity with regular trees (LDR), low diversity with clustered trees (LDC), and high density of Musaceae (HDM).

**Fig 5:**
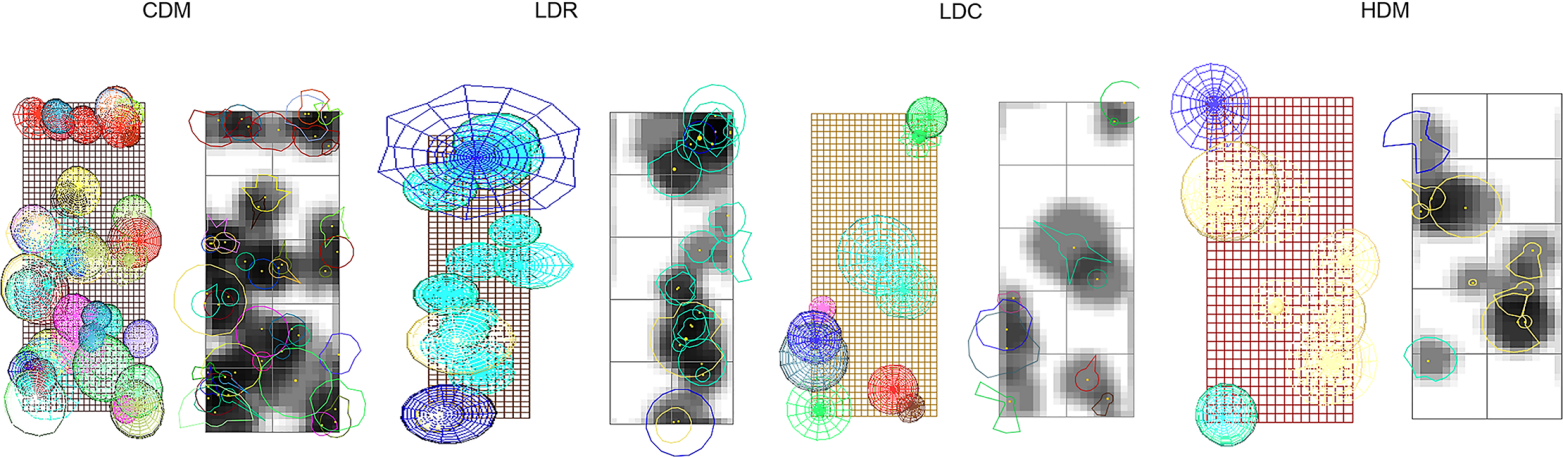
Shade projection from the simulated horizontal and vertical structure of a given plot illustrative of each type. The simulations of structure were made with SExI-FS software and the shade projection simulations were made with Shademotion software. The four types were: complex diversified multistrata (CDM), low diversity with regular trees (LDR), low diversity with clustered trees (LDC), and high density of Musaceae (HDM).

Fig 4 presents the simulated types of our typology and Fig 5 the shade projection in each type. The simulations were made using SExI-FS and Shademotion software. Fig 5 highlights the variability of light availability in each type. Light availability (transmitted radiation, fPARt) between the types was reduced by 52.4% between the CDM type with the least light availability (680.4 μmol m^-2^ s^-1^) and the HDM type with the greatest light availability (1400.6 μmol m^-2^ s^-1^). fPARt was not significantly different between the CDM (680.4 μmol m^-2^ s^-1^) and LDR (820.3 μmol m^-2^ s^-1^) types, but was least in the CDM type.

The LDC (1200.8 μmol m^-2^ s^-1^) and HMD (1400.6 μmol m^-2^ s^-1^) types had the highest fPARt, with a value in the HMD type that was significantly greater than the value in the LDC type. This tendency is visible in Fig 5, where the simulated shade projections were more intense in the CDM type, and gradually decreased up to the HDM type.

## Discussion

In this paper, we present a typology of agroforest systems in Colombian Amazonia. These systems have never been described in the literature. Specifically, as we worked in a zone considered as a deforestation front in Colombian Amazonia, which is also a post-conflict area, we set out to document the detailed characteristics of such systems, which are going to be of key importance in the future. Indeed, cacao cropping systems play a key role in post-conflict resolution: cacao is the crop for peace, i.e. a legal crop to replace illegal crops. A precise description enables an evaluation of potential, and is also a first step towards thinking about sustainable management in the context of the study zone.

The typology we built was based on classic variables of shade tree species composition and vertical structure [12,15,37], and this typology also included some original variables: horizontal structure of shade trees [38,39] and light availability, i.e. light transmitted to the cacao trees in the understorey. Using these 28 variables enabled us to identify 4 types of agroforest systems for which we detailed the characteristics: complex diversified multistrata (CDM), low diversity with regular trees (LDR), low diversity with clustered trees (LDC), and high density of Musaceae (HDM). The differences concerning the types identified mainly concerned, (i) shade tree species diversity, (ii) shade tree species density and structure, and (iii) light availability for the cacao trees, related to shade canopy structure and density [18].

### Composition and tree species richness in the agroforest systems of Colombian Amazonia

In addition to being productive systems, cacao agroforest systems also achieve biodiversity conservation goals. Cacao tree dynamics need shade, and cacao trees are generally grown in complex cropping systems, i.e. with a great diversity of forest or introduced plant species. Thus, in the different cacao production regions around the world, these cacao cropping systems display high species diversity, associated with the presence of shade trees, whose species and density vary between systems. For example, there have been reports of Brazilian cacao agroforests with 280 tree species [40], or 237 species for cacao agroforests in Ivory Coast [41] and in Indonesia [42], 205 species for cacao agroforests in Cameroon [15,43], 196 species for cacao agroforests in Ghana [44] and around 53 species of trees in Costa Rican cacao agroforests [12]. Conservation of the plant diversity in these agroforest systems is associated with management practices for the generation of shade for cacao trees, but it is also associated with the generation of different ecosystem services associated with the presence of shade trees in such systems [24,45].

In our study, the cacao agroforest systems studied in Colombian Amazonia also displayed a high diversity of tree species, with 127 tree species found. We also found tree species exclusive to each type of our typology, which were species found in one or two plots out of selected plots. We can almost say that each time we inventoried a new plot, we found new species. These ecosystems are extremely rich. We believe that in this study we are far from having assessed the total biodiversity potential of these systems, which are close to a tropical, rich forest ecosystem and specifically to a national park with high conservation value. More efforts should be made to characterise these systems, and especially their potential in terms of biodiversity conservation–also taking into account other plant and animal biodiversity–in these agroforest ecosystems, in order to promote them as a green alternative in the Colombian context. In this study, we sampled about 20% of the total area of cacao agroforest plantations. However, as we worked with plots in 50 different plantations with different structural characteristics, we could assume that this data set covered a large range of the existing structural variability. We took a first step towards providing reliable information on the biodiversity in these isolated Colombian systems; information which has been quite elusive to date [6].

However, in the agroforests studied we also found that the different types of cacao agroforests identified displayed different levels of species diversity, with a critical reduction in species richness from the “complex diversified multistrata” type (CDM, a mean of 12.92 species per plot over the 13 plots), which was the most diverse, to the “High Density of Musaceae type (HMD, a mean of 3 species per plot over the 9 plots). In fact, the 13 plots–out of 50—of the CDM type accounted for the mean of the number of species found in the studied agrosystems. We could conclude that most of the plots we studied were actually undergoing biodiversity erosion. This statement may be a cause for worry, as we know that post-conflict regions may face complicated issues in relation to biodiversity concerns. For example, in Peru, intensive agricultural activities have led to high deforestation rates and catastrophic climate events [6]. In another context, in Costa Rica, in order to cope with a severe Frosty Pod Rot epidemic, the development by projects of cacao agroforest systems led to the establishment of cacao trees associated with banana plants, or with a specialised shade of just one or two tree species [24].

One challenging investigation prospect will be to monitor and encourage the conservation of tree species diversity in cacao agroforest systems during the development of cacao cropping systems in Colombian Amazonia. Incentives promoting forest-friendly land use systems may be a possible strategy for preserving diversity in cacao agroforest systems there.

Trees in agroforest systems are related to a range of ecosystem services [45]. Other perpectives would be to characterise the ecosystem services related to the existence of these trees in the studied systems taking into account the different types found, as for example the carbon stock in the different types. Improving carbon storage may also be another reason for maintaining tree species in cacao agroforest systems. Trees are also related to production services in these systems: food, timber, etc. Working also on the potential economic value of maintaining trees in these systems will also be a strategy for preserving tree diversity in the studied cacao agroforest systems.

Lastly, regarding tree species diversity in the studied plots, in this paper we gave the detailed characteristics of the study systems, which are key systems in the study region. As we have already said, this first precise description is necessary in order to evaluate the potential, but also to set up appropriate management in the context of the study zone. However, we did not study the causes or drivers of the differences found between the types described. It is reasonable to suspect an internal plot factor, namely variation in management practices between plots, or an external plot factor such as landscape influence. Indeed, the study zone is located near the Chiribiquete national park, in the heart of Amazonia, which is a prioritary conservation zone. The influence of (i) farmer management in the plots and (ii) the vicinity of the Chiribiquete national park on tree species diversity remains to be investigated.

### Light availability in the different types in relation to stand structure

Given that the optimum transmitted radiation for the good ecophysiological development of cacao trees is 400 μmol m^-2^ s^-1^, we concluded that all the types described were compatible with good ecophysiological development, and were thus propitious to cacao agronomic production. Even though we found a reduction of 52.4% between the CDM type with the least light availability (680.4 μmol m^-2^ s^-1^) and the HDM type with the greatest light availability (1400.6 μmol m^-2^ s^-1^), the CDM light availability computed from our data was greater than the minimum fPARt needed: 400 μmol m^-1^ s^-2^ [19]. In other words, even the most shaded plots (belonging to the CDM type, which were also those that harboured greater species diversity) had enough light for good ecophysiological development of the cacao trees.

This finding is very important, as ensuring optimum light availability for cacao trees is one of the main drivers of the reduction in shade trees in cacao agroforest systems. Cacao is an understorey species, but its productivity is affected by heavy shading [46]. Reducing shade tree density (hence shade tree diversity) is also believed to reduce the rate of disease incidence. We found that this was not the case in Costa Rica, where an optimised spatial organisation of shade trees was more effective in controlling Frosty Pod Rot than a reduced shade tree density [24,38].

In the study region of Colombian Amazonia, which is characterised by a tropical humid climate, with high cloud-cover levels, characterising light availability for cacao trees in the understorey is a prerequisite for designing efficient canopy structures that optimise cacao ecophysiological development and productivity. Our characterisation of light availability based on static (one moment, one season) measurement, but also a dynamic modelling approach, might be strengthened by long-term studies designed to characterise long-term light availability in this region. We focused on light availability as it is a key factor influencing cacao tree ecophysiological development, hence production, but one important prospect will also be to assess long-term cacao yields in the different types to see, for example, the impact of the differences in light availability between CDM and HDM on long-term cacao yield. However, it may also be important to take into account cacao genetic variability and the age of the plantation when assessing this long-term cacao yield in the different types, as genetic variability and cacao tree age are also important factors influencing cacao yield. We believe that such work will be feasible in our study plots as the farmers, being in the same organisation, are committed to using commercial cacao planting material approved by the National Cacao Commission of Colombia and have registered plantation years. Lastly, in addition to light availability, which is linked to canopy structure, we may also consider losses due to pest and disease attacks in each type, which are also related to shade density and distribution [38]

## Conclusion

In this paper, we present a typology of agroforest systems in Colombian Amazonia. We set out to provide and enhance knowledge about these important systems, which have never been described in the literature. Our typology is based on variables for shade trees species composition and vertical structure, which are classically used in the typological approach applied to agroforest systems. In addition, our typology includes some original variables: horizontal structure of shade trees and light availability, i.e. light transmitted to the cacao trees in the understorey. These original variables are crucial to understanding the functioning of these systems. Indeed, the canopy structure influences light distribution and availability for cacao.

The typology we built identifies 4 types of agroforest system: complex diversified multistrata (CDM), low diversity with regular trees (LDR), low diversity with clustered trees (LDC), and a high density of Musaceae (HDM). The differences concerning the identified types mainly concerned, (i) shade tree species diversity, (ii) shade tree species density and structure, (iii) light availability for cacao trees.

Cacao agroforest systems in Colombian Amazonia are actually productive agricultural systems achieving biodiversity conservation goals. These systems may be considered as biodiversity-friendly land use. They could be a way of including biodiversity preservation in rural development plans. However, as has been seen in other post-conflict regions, the development of promoted legal crops in intensive agricultural systems has led to deforestation, biodiversity erosion and has been associated with catastrophic climate events. In the typology we have presented, we already found that most of the plots we studied were actually undergoing biodiversity erosion. We also found that all the types described were compatible with the good ecophysiological development of cacao trees, and were thus propitious to their agronomic production. Even the most diverse type, CDM with the least light availability (680.4 μmol m-2 s-1) may be compatible with the good ecophysiological development of cacao trees. One challenging prospect in the post-conflict context of Colombian Amazonia will be to monitor and encourage the conservation of tree species diversity in cacao agroforest systems during the development of these cropping systems. This may be achieved through incentives to promote the preservation of tree diversity in cacao agroforest systems as a forest-friendly land use system enhancing sustainable peace building in Columbia.

## Supporting information

S1 File The set of data used for the analyses. Raw data of tree species abundance, frequency, dominance and basal area in the study plots, used to compute the Importance Value Index (IVI) and the Physiognomic Predominance Index PPI.
(XLSX)

S2 File List of all the species found in the study plots. (XLSX)

S3 File The geographical coordinates of the different plots. (XLSX)

## References

[1] ICCO (2017) International Cocoa Organization. Quarterly Bulletin of Cocoa Statistics, Vol. XL, No. 1, Cocoa year 2013/14.

[2] Somarriba E, Beer J, Orihuela JA, Andrade H, Cerda R, DeClerck F, et al (2012) Mainstreaming agroforestry in Latin America; Nair PKR GD, editor. New York: Springer.

[3] Phillips-Mora W, Aime MC, Wilkinson MJ (2007) Biodiversity and biogeography of the cacao (Theobroma cacao) pathogen Moniliophthora roreri in tropical America. Plant Pathology 56: 911–922.

[4] Sierra DC (2016) El cacao como producto lider en la substitution de cultivos ilicitos en el proceso de posconflicto Facultad de Relaciones Internacionales, Estrategia y Seguridad Programa de Relaciones Internacionales y Estudios Políticos. Universidad Militar Nueva Granada. 29 p.

[5] FEDECACAO (2017) Federación Nacional de Cacaoteros. Departamento de estadistica. Colombia. http://www.fedecacao.com.co/portal/index.php/es/2015-02-12-17-20-59/nacionales.

[6] Baptiste B, Pinedo-Vasquez M, Victor H, Gutierrez-Velez, Andrade GI, Vieira P, et al (2017) Greening peace in Colombia. Nature Ecology and Evolution 1(4). 10.1038/s41559-017-0102 28812667

[7] Sanchez-Cuervo AM, Aide TM (2013) Consequences of the Armed Conflict, Forced Human Displacement, and Land Abandonment on Forest Cover Change in Colombia: A Multi-scaled Analysis. Ecosystems 16: 1052–1070.

[8] Tomassone R, Dervin C, Masson JP (1993) Biometrie. Modelisation de phenomenes biologiques. Issy-les-Moulineaux, FRANCE: Elsevier Mason SAS. 553 p.

[9] Johns ND (1999) Conservation in Brazil's chocolate forest: The unlikely persistence of the traditional cocoa agroecosystem. Environmental Management 23: 31–47. 9817770 10.1007/s002679900166

[10] Moguel P, Toledo VM (1999) Biodiversity conservation in traditional coffee systems of Mexico. Conservation Biology 13: 11–21.

[11] Rice RA, Greenberg R (2000) Cacao cultivation and the conservation of biological diversity. Ambio 29: 167–173.

[12] Deheuvels O, Avelino J, Somarriba E, Malezieux E (2012) Vegetation structure and productivity in cocoa-based agroforestry systems in Talamanca, Costa Rica. Agriculture Ecosystems & Environment 149: 181–188.

[13] Somarriba E, Lachenaud P (2013) Successional cocoa agroforests of the Amazon—Orinoco -Guiana shield. Forests, Trees and Livelihoods 22: 51–59.

[14] Oke DO, Odebiyi KA (2007) Traditional cocoa-based agroforestry and forest species conservation in Ondo State, Nigeria. Agriculture Ecosystems & Environment 122: 305–311.

[15] Sonwa DJ, Weise SF, Nkongmeneck BA, Tchatat M, Janssens MJJ (2017) Structure and composition of cocoa agroforests in the humid forest zone of Southern Cameroon. Agroforestry Systems 91: 451–470.

[16] Kessler M, Kessler PJA, Gradstein SR, Bach K, Schmull M, Pitopang R. (2005) Tree diversity in primary forest and different land use systems in Central Sulawesi, Indonesia. Biodiversity and Conservation 14: 547–560.

[17] Bisseleua HBD, Fotio D, Yede, Missoup AD, Vidal S (2013) Shade Tree Diversity, Cocoa Pest Damage, Yield Compensating Inputs and Farmers' Net Returns in West Africa. Plos One 8. 10.1371/journal.pone.0056115 PMC3592863 23520451

[18] Mariscal MJ, Martens SN, Ustin SL, Chen JQ, Weiss SB, Roberts DA (2004) Light-transmission profiles in an old-growth forest canopy: Simulations of photosynthetically active radiation by using spatially explicit radiative transfer models. Ecosystems 7: 454–467.

[19] Avila-Lovera E, Coronel I, Jaimez R, Urich R, Pereyra G, Araque O, et al (2016) ECOPHYSIOLOGICAL TRAITS OF ADULT TREES OF CRIOLLO COCOA CULTIVARS (THEOBROMA CACAO L.) FROM A GERMPLASM BANK IN VENEZUELA. Experimental Agriculture 52: 137–153.

[20] Almeida AAF, Gomes FP, Araujo RP, Santos RC, Valle RR (2014) Leaf gas exchange in species of the Theobroma genus. Photosynthetica 52: 16–21.

[21] Jezeer RE, Verweij PA, Santos MJ, Boot RGA (2017) shaded Coffee and Cocoa-Double Dividend for Biodiversity and Small-scale Farmers. Ecological Economics 140: 136–145.

[22] Charbonnier F, le Maire G, Dreyer E, Casanoves F, Christina M, Dauzat J, et al (2013) Competition for light in heterogeneous canopies: Application of MAESTRA to a coffee (Coffea arabica L.) agroforestry system. Agricultural and Forest Meteorology 181: 152–169.

[23] Gao LB, Xu HS, Bi HX, Xi WM, Bao B, Wang X et al (2013) Intercropping Competition between Apple Trees and Crops in Agroforestry Systems on the Loess Plateau of China. Plos One 8. 10.1371/journal.pone.0070739 PMC3723670 23936246

[24] Ngo Bieng MA, Gidoin C, Avelino J, Cilas C, Deheuvels O, Wery J (2013) Diversity and spatial clustering of shade trees affect cacao yield and pathogen pressure in Costa Rican agroforests. Basic and Applied Ecology 14: 329–336.

[25] Frazer GW, Canham CD, Lertzman KP (1999) Gap Light Analyzer (GLA), Version 2.0: Imaging Software to Extract Canopy Structure and Gap Light Transmission Indices from True-Colour Fisheye Photographs, User’s Manual and Program Documentation. The Institute of Ecosystem Studies, Millbrook, NY.

[26] Colwell RK, Elsensohn JE (2014) EstimateS turns 20: statistical estimation of species richness and shared species from samples, with non-parametric extrapolation. Ecography 37: 609–613.

[27] Bharathi S, Prasad AGD (2017) Diversity, population structure and regeneration status of arboreal species in the four sacred groves of Kushalnagar, Karnataka. Journal of Forestry Research 28: 357–370.

[28] Rangel JOC, Avella A (2011) Oak forests of Quercus humboldtii in the Caribbean region and distribution patterns related with environmental factors in Colombia. Plant Biosystems 145: 186–198.

[29] Clark PJ, Evans FC (1954) Distance to nearest neighbor as a measure of spatial relationships in populations. Ecology 35: 445–453.

[30] Zhang DS, Zhang LZ, Liu JG, Han S, Wang Q, Evers J, et al (2014) Plant density affects light interception and yield in cotton grown as companion crop in young jujube plantations. Field Crops Research 169: 132–139.

[31] Quesada F, Somarriba E, Malek M (2007) ShadeMotion 3.0: Software para calcular la cantidad de horas de sombra que proyectan un conjunto de árboles sobre un terreno. 31 p.

[32] Harja D, Vincent G (2008) Spatially Explicit Individual-based Forest Simulator—User Guide and Software. World Agroforestry Centre (ICRAF) and Institut de Recherche pour le Développement (IRD). 93 p.

[33] Di Rienzo JA, Casanoves F, Balzarini MG, Gonzalez L, Tablada M, Robledo CW (2017) InfoStat versión. Grupo InfoStat, FCA, Universidad Nacional de Córdoba, Argentina.

[34] Lê S, Josse J, Husson F (2008) FactoMineR: An R Package for Multivariate Analysis. Journal of Statistical Software 25: 1–18.

[35] Dray S, Dufour AB (2007) The ade4 package: Implementing the duality diagram for ecologists. Journal of Statistical Software 22: 1–20.

[36] Team RDC (2016) R: A language and environment for statistical computing. R. In: Foundation for Statistical Computing V, Austria. ISBN 3-900051-07-0, editor.

[37] Sonwa DJ, Nkongmeneck BA, Weise SF, Tchatat M, Adesina AA, et al (2007) Diversity of plants in cocoa agroforests in the humid forest zone of Southern Cameroon. Biodiversity and Conservation 16: 2385–2400.

[38] Gidoin C, Avelino J, Deheuvels O, Cilas C, Bieng MAN (2014) Shade tree spatial structure and pod production explain frosty pod rot intensity in cacao agroforests, Costa Rica. Phytopathology 104: 275–281. doi: 10.1094/PHYTO-07-13-0216-R 24168046 10.1094/PHYTO-07-13-0216-R

[39] Gidoin C, Babin R, Beilhe LB, Cilas C, ten Hoopen GM, Ngo Bieng MA (2014) Tree Spatial Structure, Host Composition and Resource Availability Influence Mirid Density or Black Pod Prevalence in Cacao Agroforests in Cameroon. Plos One 9. 10.1371/journal.pone.0109405 PMC4196851 25313514

[40] Sambuichi RHR, Vidal DB, Piasentin FB, Jardim JG, Viana TG, Menezes AA, et al (2012) Cabruca agroforests in southern Bahia, Brazil: tree component, management practices and tree species conservation. Biodiversity and Conservation 21: 1055–1077.

[41] Tondoh JE, Kouamé FNg, Martinez Guéi A, Sey B, Wowo Koné A, et al (2015) Ecological changes induced by full-sun cocoa farming in Côte d’Ivoire. Global Ecology and Conservation 3: 575–595.

[42] Steffan-Dewenter I, Kessler M, Barkmann J, Bos MM, Buchori D, Erasmi S, et al (2007) Tradeoffs between income, biodiversity, and ecosystem functioning during tropical rainforest conversion and agroforestry intensification. Proceedings of the National Academy of Sciences of the United States of America 104: 4973–4978. doi: 10.1073/pnas.0608409104 17360392 10.1073/pnas.0608409104 PMC1829249

[43] Mbolo MMA, Zekeng JC, Mala WA, Fobane JL, Chimi C, Ngavounsia T, et al (2016) The role of cocoa agroforestry systems in conserving forest tree diversity in the Central region of Cameroon. Agroforestry Systems 90: 577–590.

[44] Asase A, Ofori-Frimpong K, Ekpe PK (2010) Impact of cocoa farming on vegetation in an agricultural landscape in Ghana. African Journal of Ecology 48: 338–346.

[45] Tscharntke T, Clough Y, Bhagwat SA, Damayanti B, Faust H, Hertel D, et al (2011) Multifunctional shade-tree management in tropical agroforestry landscapes—a review. (Special Issue: The Future of Agri-Environment Schemes.). Journal of Applied Ecology 48: 619–629.

[46] Zuidema PA, Leffelaar PA, Gerritsma W, Mommer L, Anten NPR (2005) A physiological production model for cocoa (Theobroma cacao): model presentation, validation and application. Agricultural Systems 2005 84: 2, 195–225 many ref.

